# Divergent activity of the gonadotropin-releasing hormone receptor gene promoter among genetic lines of pigs is partially conferred by nuclear factor (NF)-B, specificity protein (SP)1-like and GATA-4 binding sites

**DOI:** 10.1186/s12958-016-0170-0

**Published:** 2016-06-29

**Authors:** Emily A. McDonald, Jacqueline E. Smith, Rebecca A. Cederberg, Brett R. White

**Affiliations:** Laboratory of Reproductive Biology, Department of Animal Science, Institute of Agriculture and Natural Resources, University of Nebraska-Lincoln, Lincoln, NE USA; Present address: Center for International Health Research, Rhode Island Hospital, Providence, RI USA; Present address: Stowers Institute for Medical Research, Kansas City, MO USA

**Keywords:** GnRH receptor, Transcriptional regulation, NF-κB, GATA-4, SP1-like factor, Porcine, Single nucleotide polymorphism, Ovulation rate, Anterior pituitary, Gonadotrope

## Abstract

**Background:**

Binding of gonadotropin-releasing hormone (GnRH) to its receptor (GnRHR) on gonadotropes within the anterior pituitary gland is essential to reproduction. In pigs, the GnRHR gene is also located near a genetic marker for ovulation rate, a primary determinant of prolificacy. We hypothesized that pituitary expression of the GnRHR gene is alternatively regulated in genetic strains with elevated ovulation rates (Chinese Meishan and Nebraska Index) vs. standard white crossbred swine (Control).

**Methods:**

Luciferase reporter vectors containing 5118 bp of GnRHR gene promoter from either the Control, Index or Meishan swine lines were generated. Transient transfection of line-specific, full length, deletion and mutation constructs into gonadotrope-derived αT3-1 cells were performed to compare promoter activity and identify regions necessary for divergent regulation of the porcine GnRHR gene. Additionally, transcription factors that bind the GnRHR promoter from each line were identified with electrophoretic mobility shift assays (EMSA).

**Results:**

Dramatic differences in luciferase activity among Control, Index and Meishan promoters (19-, 27- and 49-fold over promoterless control, respectively; *P* < 0.05) were established. A single bp substitution (−1690) within a previously identified upstream enhancer (−1779/−1667) bound GATA-4 in the Meishan promoter and the p52/p65 subunits of nuclear factor (NF)-κB in the homologous Control/Index promoters. Transient transfection of vectors containing block replacement mutations of either the GATA-4 or NF-κB binding sites within the context of their native promoters resulted in a 50 and 60 % reduction of luciferase activity, respectively (*P* < 0.05). Furthermore, two single-bp substitutions in the Meishan compared to Control/Index promoters resulted in binding of the p52 and p65 subunits of NF-κB and a specificity protein 1 (SP1)-like factor (−1235) as well as GATA-4 (−845). Vectors containing the full-length Meishan promoter harboring individual mutations spanning these regions reduced luciferase activity by 25 and 20 %, respectively, compared to native sequence (*P* < 0.05).

**Conclusions:**

Elevated activity of the Meishan GnRHR gene promoter over Control/Index promoters in αT3-1 cells is partially due to three single nucleotide polymorphisms resulting in the unique binding of GATA-4 (−1690), the p52/p65 subunits of NF-kB in combination with a SP1-like factor (−1235), and GATA-4 (−845).

**Electronic supplementary material:**

The online version of this article (doi:10.1186/s12958-016-0170-0) contains supplementary material, which is available to authorized users.

## Background

The hypothalamic decapeptide, gonadotropin-releasing hormone (GnRH), binds to its cognate receptor (GnRHR) on the surface of gonadotrope cells within the anterior pituitary gland, stimulating the synthesis and secretion of the gonadotropins, follicle stimulating hormone (FSH) and luteinizing hormone (LH) [1, 2]. The gonadotropins subsequently act on the gonads to trigger secretion of steroid hormones, which feedback at the level of the hypothalamus and anterior pituitary gland to regulate GnRH and gonadotropin levels, respectively. Binding of GnRH to its seven transmembrane, G-protein-coupled receptor activates multiple signal transduction cascades, ultimately resulting in up-regulation of the genes that encode the common α- and unique β-subunits of FSH and LH [3, 4] as well as the GnRHR itself [5]. Therefore, the interaction between GnRH and GnRHR represents a crucial point for regulation of reproductive function in mammals. Furthermore, the porcine GnRHR gene is located on chromosome 8, in close proximity to a quantitative trait locus for ovulation rate, a primary determinant of litter size [6]. Consistent with this, a C/G substitution in the 3’ untranslated region was shown to be significantly associated with ovulation rate [7]. Consequently, the GnRHR gene represents both a physiological and positional candidate for genes influencing prolificacy in pigs.

The GnRHR gene promoter has been extensively studied in the mouse, rat, human and sheep [8, 9]. In the mouse, gonadotrope-specific expression is conferred by 500 bp of GnRHR gene promoter [10] comprised of binding sites for steroidogenic factor 1 (SF1), activator protein 1 (AP1) and a GnRHR activating sequence (GRAS) [11, 12], although pituitary homeobox (Pitx)-1 and a member of the LIM homeodomain family, Lhx3, have also been implicated [13, 14]. Additionally, protein kinase C (PKC) activation of AP1 is critical for GnRH responsiveness of the murine GnRHR gene promoter [15], whereas the GRAS binding site acts in conjunction with a downstream activin regulatory element to control activin responsiveness [16]. Subsequent studies revealed that the GRAS element binds a complex of transcription factors including AP1, Smad3 and 4 and FOXL2, a member of the forkhead family of transcription factors [17]. Furthermore, an enhancer element, sequence underlying responsiveness to GnRH (SURG)-1 [18], binds octamer transcription factor-1 (OCT1) and nuclear factor (NF)-Y [19] for basal and maximal GnRH stimulation of GnRHR gene transcription. A proximal homeodomain (Hbox) binding motif also binds OCT1, indicating the transcription factor acts at multiple TAAT sites to direct basal expression [20]. Interestingly, the CLOCK and BMAL1 drive activity of the murine GnRHR promoter through their interaction with E-box enhancer sequences [21]. The importance of protein kinase A (PKA) signaling was also illustrated by the role of a cAMP responsive element (CRE) in activation of the GnRHR gene [22].

The rat and mouse GnRHR gene promoters appear consistent, both containing SF1, AP1 and GRAS binding sites [23]. However, the GRAS element in the rat GnRHR promoter harbors an A → G bp alteration, dramatically reducing effectiveness of this binding site [24]. Furthermore, the rat promoter contains CRE-like and SF1 adjacent protein (SAP) binding sites involved in basal activity [25] and a GnRHR specific enhancer (GnSE) [24] that interacts with GATA factors and the LIM-related factors, Isl-1 and Lhx3, to promote maximal basal activity [25]. Although activity of the human GnRHR promoter in cell lines of non-gonadotrope origin and in response to hormones have been characterized [8], investigation into gonadotrope-specific activity has only elucidated a SF1 binding site [26]. Additional studies revealed that AP1 confers down-regulation following GnRH stimulation [27] and OCT1 serves as a strong constitutive repressor [28]. Elements conferring gonadotrope-specific expression of the ovine GnRHR gene remain to be elucidated; however, Duval and coworkers [29] reported a SF1 binding site that mediates basal expression.

Our laboratory has previously shown that gonadotrope-specific activity of the porcine GnRHR gene is partially conferred by a SF1 binding site positioned within a 112-bp upstream enhancer (−1779/−1667), as well as two additional SF1 and one retinoid X receptor (RXR) binding sites located within 315 bp of proximal promoter [30]. In order to compare transcriptional regulation of the GnRHR gene among pig lines with divergent ovulation rates, we constructed luciferase reporter constructs containing 5118 bp of 5’ flanking sequence from three genetic lines of swine: a Control white-crossbred line; a Nebraska Index line selected for over 14 generations based on an index of ovulation rate and embryonic survival [31]; and the Chinese Meishan breed, a line with increased prolificacy over white-crossbred lines, largely due to a greater ovulation rate [32, 33]. Previously, our laboratory has shown that anterior pituitary levels of GnRHR mRNA were highest in Meishan, intermediate in Index and lowest in Control [34]. Herein, we demonstrate differential activity among these line-specific GnRHR promoters utilizing transient transfections assays in gonadotrope-derived αT3-1 cells. In addition, we identified three bp substitutions at −1690 (T → C), −1235 (C → G) and −845 (G → T) of proximal promoter that allow GATA-4, the p52 and p65 subunits of nuclear factor (NF)-κB as well as a specificity protein (SP)1-like factor, and GATA-4, respectively, to preferentially bind the Meishan compared to Index or Control GnRHR gene promoters.

## Methods

Experiments involving the use of recombinant DNA have been approved by the UNL Institutional Biosafety Committee under Protocol ID # 12 entitled: Functional Analysis of GnRHR I and II in Swine. The UNL Radiation Safety Office has approved the use of isotopes in the following experiments via AU License # I-387.

## Materials

The antibody directed against the p65 subunit of NF-κB (catalog no. PC137) was purchased from Calbiochem (La Jolla, CA), the antibodies specific for the p52 subunit of NF-κB (catalog no. 06–413), SP1 (catalog no. 07–645), SP3 (catalog no. 07–107) were from Upstate (Charlottesville, VA), the specific antibodies for the p50 subunit of NF-κB (catalog no. sc-114X), SP1 (catalog no. sc-59X), SP2 (catalog no. sc-643X), SP4 (catalog no. sc-13019X), GATA-1 (catalog no. sc-1234X), GATA-2 (catalog no. sc-9008X), GATA-4 (catalog no. sc-1237) and normal rabbit IgG (catalog no. sc-2027) were obtained from Santa Cruz Biotechnology, Inc. (Santa Cruz, CA). For experiments using EMSA, competitive oligonucleotides containing consensus binding sites for activator protein (AP)2, NF-κB, SP1 or GATA were synthesized by Integrated DNA Technologies (Coralville, IA; Table 1).

**Table 1:** Sense strand of EMSA oligos^a^

Name	Sequence
AP2 consensus^b^	5’-GATCGAACTGACCGCCCGCGGCCCGT-3’
GATA consensus^b^	5’-CACTTGATAACAGAAAGTGATAACTCT-3’
GR consensus^b^	5’-AGAGGATCTGTACAGGATGTTCTAGAT-3’
NF1 consensus^b^	5’-TTTTGGATTGAAGCCAATATGATA-3’
NF-κB consensus^b^	5’-AGTTGAGGGGACTTTCCCAGGC-3’
SP1 consensus^b^	5’-AATCGATCGGGGCGGGGCGAG-3’
C/I −855/−835^c^	5’-GCATACAAAG***G***GATATAAACA-3’
M −853/−833^c^	5’-GCATACAAAG***T***GATATAAAC-3’
C/I −1245/−1225^c^	5’-AGCTTCCTCA***C***GGCCTGGATG-3’
M −1243/−1223^c^	5’-AGCTTCCTCA***G***GGCCTGGATG-3’
C/I −1700/−1680^c^	5’-AACCCCATAT***T***TCCACTGAGA-3’
M −1676/−1656^c^	5’-AACCCCATAT***C***TAGGCACTAA-3’

^a^The complement strand was annealed for each oligonucleotide prior to use in gel shift assays

^b^AP2, activator protein 2; GR, glucocorticoid receptor; NF1, nuclear factor 1; NF-B, nuclear factor-κB; SP1, specificity protein 1

^c^Base pair substitutions between the different breed promoters are in bold italics

### Plasmids

Using primers specific for the porcine GnRHR gene promoter originally isolated from the Control line [30], we sequenced promoters from genomic DNA of the Meishan and Index lines. Full-length GnRHR gene promoters (−5118) from the three genetic pig lines were sub-cloned into the pGL3 basic reporter vector (Promega Corp., Madison, WI). Studies involving constructs containing progressively less 5’ flanking sequence of the GnRHR gene promoter for all three lines of pigs were generated by restriction endonuclease digestion of vectors containing the full-length GnRHR gene promoter for each line and subsequent intramolecular ligation of the remaining vector backbone (*PvuII, SpeI* and *BlpI)*. The promoter “swap” vectors containing full-length GnRHR gene promoter with the region from the −1915 to −1431 bp exchanged between Control and Meishan promoters was constructed from vectors containing 5118 bp of native sequence. Restriction endonuclease digestion of the internal 484 bp and subsequent ligation of the corresponding region for the promoter of the other line of swine was performed. Overlap extension PCR mutagenesis was performed through two rounds of PCR in order to specifically mutate the binding element of interest [35]. The first round of PCR utilized primers replacing the binding site of interest with a restriction site, and the second round used product from the first round as template to anneal and replicate the mutated element and flanking sequence (Table 2). The mutation of the SF1 binding sites located at −179/−171 in each of the promoters was performed with the same set of primers and generated a *NotI* restriction enzyme site. The −MμGATAUEpGL3, −MμNF-κBpGL3, −MμSP1pGL3 and −MμGATA4pGL3 plasmids were composed of 5118 bp of 5’ flanking sequence for the Meishan GnRHR gene with individual elements mutated to contain either a *SpeI* (−MμGATAUEpGL3), *NsiI* (−MμNF-κBpGL3), *SpeI* (−MμSP1pGL3) or *PstI* (−MμGATA4pGL3) site. The −Mμ1240pGL3 plasmid contained a double mutation of the NF-κB and SP1 sites discussed above. The −CμNF-κBpGL3 plasmid was made by substituting the NF-κB site for *EcoRI.* To verify that the correct mutations had been introduced, vectors were sequenced before use in transient transfection experiments. The vector used as a control for transfection efficiency in all experiments contained the Rous Sarcoma Virus (RSV) promoter fused to the cDNA encoding β-galactosidase (RSV-βgal, Stratagene, La Jolla, CA). A midi plasmid preparation kit (Qiagen, Valencia, CA) was used to isolate transfection quality DNA.

**Table 2:** Primers used to generate reporter vectors

Name	Sequence
−5118pGL3 F	5’-CAGACAATTAGATTCCAGGGC-3’
Promoter R	5’-TCCTTCCCCAACTGATGTAG-3’
μSF1pGL3 F^a^	5’-AAGTACACAAAACAAGTTGCGGCCGGCTCTTTCACATTAAATATA-3’
proximal A OF	5’-GTTATGTGGAAGAGCCGGTG-3’
proximal OR	5’-CTTTATGTTTTTGGCGTCTTCC-3’
MμGATAUEpGL3F^a^	5’-TTGCAGAAACCTAACCCCACTAGTAGGCACTAATCCAGTGTC-3’
CμNF-κBpGL3 F^a^	5’-TTGGCTTGCAGAAACCTAGAATTCTATTTCCACTGAGAGCAA-3’
distal OF	5’-CAGAGAATGCTATTGCTCTC-3’
distal OR	5’-GTGTAAGTGTTGGAACCACATC-3’
Mμ1240pGL3 F^a^	5’-CATAGCACCAAGGAAGCTATGCATACTAGTGGATGATACTGTGTGCAG-3’
proximal B OF	5’-AGGCACTAATCCAGTGTCTGC-3’
proximal OR	5’-CTTTATGTTTTTGGCGTCTTCC-3’
MμSP1pGL3 F^a^	5’-ACCAAGGAAGCTTCCTCAACTAGTGGATGATACTGTGTGCAG-3’
MμNF-κBpGL3 F^a^	5’-CATAGCACCAAGGAAGCTATGCATGGGCCTGGATGATACTGT-3’
MμGATA4pGL3 F^a^	5’-ATTAGATTGCATACAAAGCTGCAGAAACAAATATTCATATTA-3’
proximal C OF	5’-TACTCCTCTTGATTTCTGACTC-3’
proximal OR	5’-CTTTATGTTTTTGGCGTCTTCC-3’

^a^Block replacement mutation reporter vectors were generated with outer forward (OF) or outer reverse (OR) primers as described in the Methods. The proximal OR resides in pGL3 3’ of the insert. Underlined bases indicate the new restriction enzyme digest site that replaced the transcription factor binding site in the native sequence

### Cell culture and transient transfections

Cultures of αT3-1 cells (Dr. Pam Mellon, Salk Institute, La Jolla, CA) were maintained at 37 °C in a humidified 5 % CO_2_ in air atmosphere. The αT3-1 cells were cultured in high-glucose DMEM (4.5 g/L; Mediatech, Herndon, VA) supplemented with 5 % fetal bovine serum, 5 % horse serum, 2 mM glutamine, 100 U/ml penicillin and 100 μg/ml streptomycin sulfate (Gibco, Grand Island, NY). Transient transfections were carried out using a liposome-mediated protocol (Fugene6, Roche Diagnostics Corp., Indianapolis, IN) according to manufacturer’s instructions. Briefly, 2 × 10^6^ cells were plated in 6-well culture dishes 1 d prior to transfection. Cells were transfected with a 3:1 Fugene6 to DNA ratio. A total of 1 μg of DNA, 0.9 μg of luciferase test vector and 0.1 μg of RSV-βgal control vector were used per well. Approximately 20–24 h post-transfection, cells were washed twice with ice-cold PBS and harvested with 200 μl of lysis buffer [100 mM potassium phosphate (pH 7.8), 0.2 % Triton X-100 and 1 mM dithiothreitol (DTT)]. Lysates were cleared by centrifugation at 14,000 X *g* for 2 min at 4 °C. Lysates (20 μl) were immediately analyzed according to manufacturer’s instructions for both luciferase (Promega Corp.) and β-gal (Applied Biosystems, Bedford, MA) activity using a Wallac Victor^2^ microplate reader (PerkinElmer Life Sciences, Boston, MA). Luciferase values were divided by β-gal values to adjust for transfection efficiency. The raw data for all transfections utilized in this study have been included (Additional file 1).

### EMSA

Nuclear protein extracts were obtained from approximately 2.8 × 10^8^ αT3-1 cells using the NE-PER® Nuclear and Cytoplasmic Extraction Reagents Kit (Pierce Biotechnology, Rockford, IL). The nuclear extracts were treated with protease (catalog no. P8340; Sigma Chemical Co., St. Louis, MO) and phosphatase (catalog no. 524625; Calbiochem, La Jolla, CA) inhibitor cocktail solutions to prevent enzymatic degradation of proteins. The amount of protein present in the extracts was determined using bicinchoninic acid (BCA assay, Pierce Biotechnology). Oligonucleotides were end-labeled with [γ-^32^P]ATP using T4 polynucleotide kinase (Fermentas Inc., Hanover, MD) and purified using sephadex G-25 spin columns (Amersham Biosciences Corp., Piscataway, NJ). EMSAs were completed through incubation of nuclear extracts (5 μg) in 20 μl reactions containing 4 μl of Dignam D buffer (20 mM HEPES, 20 % glycerol, 0.1 M potassium chloride, 0.2 mM EDTA and 0.5 mM DTT), 1 mM DTT, 2 μg of poly(dI•dC) (Amersham Biosciences) and, where indicated, a rabbit polyclonal antibody directed against the p65 (Calbiochem), p52 (Upstate, Lake Placid, NY), or p50 (Santa Cruz Biotechnology) subunits of NF-κB; GATA-1, -2 and -4 (Santa Cruz Biotechnology); SP1 (Upstate), SP1, SP2, SP3 and SP4 (Santa Cruz Biotechnology) or an equal mass of rabbit IgG (Santa Cruz Biotechnology) at 4 °C for 2 h. Following incubation, radiolabeled probe (100,000 cpm) and 50-fold molar excess of either homologous or heterologous unlabeled competitor was added. Where indicated, 50-fold molar excess of unlabeled oligonucleotides containing consensus binding sequences for AP2, NF-κB, SP1, glucocorticoid receptor (GR), nuclear factor (NF)-1 or GATA-4 were also added. The final reactions were incubated at 25 °C for 20 min before bound probe was separated from free at 30 mA for 1.5 h on a 5 % polyacrylamide gel that had been prerun at 100 V for 1 h in 1X TGE [25 mM Tris (pH 8.3), 190 mM glycine and 1 mM EDTA]. Gels were transferred to blotting paper, dried, and exposed to Biomax MS film (Eastman Kodak Co., Rochester, NY) for 20–24 h at −80 °C before being developed.

### Western blot

Nuclear proteins from αT3-1 cells were extracted using the Nuclear Complex Co-IP kit from Active Motif (Carlsbad, CA), quantitated with a BCA protein assay kit (Pierce) and stored at −80 °C. Protein samples (40 μg) were boiled for 5 min in a 2X reducing loading buffer (130 mM Tris pH 6.8, 4 % SDS, 0.02 % Orange G, 20 % glycerol, 100 mM DTT), cooled to room temperature (RT) and loaded onto an SDS polyacrylamide gel (PAGE) with a 5 % stacking and 10 % resolving gel. Gels were run at 40 mA for approximately 90 min and electrophoresed proteins were transferred to polyvinylidene difluoride (PVDF, Immobilon -FL, Millipore, Billerica, MA) membrane with a semi-dry electroblotter (Panther, Owl Separation Systems, Portsmouth, NH). Briefly, PVDF membrane was pre-wetted in 100 % methanol and soaked with the gel in transfer buffer (25 mM Tris pH 8.3, 192 mM glycine, 0.1 % SDS, 20 % methanol) for 15 min. The proteins were transferred at 200 mA for 1 h. Membranes were blocked with StartingBlock™ TBS buffer (Pierce) for 30 min at room temperature with agitation. Incubation of primary antibodies directed against the p50 (Santa Cruz Biotechnology), p52 (Upstate) and p65 (Calbiochem) subunits of NF-κB were performed in StartingBlock™ TBS buffer supplemented with 0.05 % Tween-20. Antibodies were used at 1:500 (p50) or 1:5000 (p52 and p65) dilutions. Blots were incubated with primary antibody overnight at 4 °C with gentle shaking. After incubation, the blots were washed four times with TBS-T (20 mM Tris pH 7.6, 137 mM sodium chloride, 0.1 % Tween-20). Each wash was performed for 5 min with gentle agitation. The secondary antibody, Alexa Fluor 680 goat anti-rabbit IgG (A21076, Invitrogen, Carlsbad, CA) was diluted 1:15,000 in StartingBlock™ TBS buffer (Pierce) supplemented with 0.01 % SDS and 0.05 % Tween-20. The incubation was performed at RT for 1 h with gentle shaking. Blots were washed four times with TBS-T for 5 min with gentle agitation. After a final rinse with TBS, blots were scanned on the 700 channel of the Odyssey Infrared Imaging System (LI-COR, Lincoln, NE) following manufacturer’s instructions.

### Statistical analysis

Data were analyzed using the general linear models (GLM) procedure of the Statistical Analysis System (SAS, version 8.2, Cary, NC). To control for transfection efficiency, the arbitrary light value for each replicate was divided by the respective β-gal value. These values were then divided by the mean of the empty vector and reported as fold activity over pGL3. All transfections were performed a minimum of three times, with samples in triplicate using different plasmid preparations for each transfection. Individual values from all the replicates were used to generate the mean ± SEM. Comparisons between pGL3 and test vectors were evaluated with Dunnett’s *t*-test. Least squares means for luciferase activity were compared among test vectors using least significant differences.

## Results

### The −5118 bp Control, Index and Meishan GnRHR promoters display divergent activity in αT3-1 cells, which is maintained upon promoter reduction to −1915 bp

**Fig. 1:**
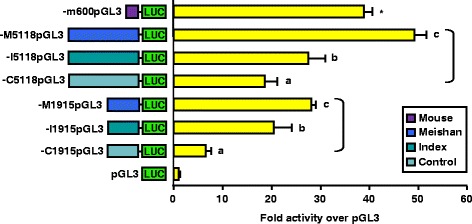
Reporter constructs containing GnRHR promoters from Meishan, Index and Control swine lines exhibit divergent activity. αT3-1 cells were transiently transfected with luciferase (LUC) reporter vectors containing either 5118 or 1915 bp of GnRHR promoter for the Meishan (−M5118pGL3 and −M1915pGL3), Index (−I5118pGL3 and −I1915pGL3) and Control (−C5118pGL3 and −C1915pGL3) lines of swine, 600 bp of the mouse GnRHR promoter (−m600pGL3), or promoterless control (pGL3). The −m600pGL3 vector was included as a positive control because it has been shown to have robust activity in this cell line. Unique letters within vectors containing the same portion of 5’ flanking sequence (−5118 or −1915) for each swine line (brackets) indicate values that are significantly different from one another (*P* < 0.05). An asterisk indicates a mean significantly different from all others (*P* < 0.05)

Transient transfection of plasmids containing the full-length GnRHR gene promoter (−5118) for either the Control (−C5118pGL3), Index (−I5118pGL3) or Meishan (−M5118pGL3) lines into gonadotrope-derived αT3-1 cells established dramatic increases (*P* < 0.05) in luciferase activity for Control (19-fold), Index (27-fold) and Meishan (49-fold) constructs over promoterless control (Fig. 1). These differences represented a 1.5- and 2.7-fold increase in luciferase activity for vectors containing the Index and Meishan promoters, respectively, over the corresponding plasmid containing the promoter from the Control line. As a positive control, we also included a luciferase reporter vector containing 600 bp of the mouse GnRHR promoter (−m600pGL3) as Clay and coworkers [11] demonstrated robust activity of this construct in αT3-1 cells. Interestingly, the 49-fold increase in luciferase activity of the Meishan reporter vector compared to promoterless controls was higher (*P* < 0.05) than that of the construct containing the mouse promoter (39-fold; Fig. 1). In summary, the differential promoter activity among the three swine lines indicates the use of divergent mechanisms for transcriptional regulation of the GnRHR gene.

Previous work in our laboratory indicated that gonadotrope-specific expression of the porcine GnRHR gene was conferred by elements located within 1915 bp of proximal promoter [30]. To determine if this region contained elements conferring line-specific expression, we reduced the 5’ flanking region for all three pig lines from −5118 to −1915 bp. Transient transfection of plasmids containing either −5118 (described above) or −1915 GnRHR promoters for the Meishan (−M1915pGL3), Index (−I1915pGL3) and Control (−C1915pGL3) lines resulted in maintenance of significant differences in luciferase activity among lines (Fig. 1). Therefore, the elements conferring line-specific expression of the porcine GnRHR gene reside within 1915 bp of proximal promoter.

### Sequence alignment of the Control, Index and Meishan GnRHR promoters identified polymorphisms at several locations within −1915 bp of promoter

Alignment of the −1915 bp Control and Meishan promoters identified 10 single bp changes and a single bp deletion (Table 3). Additionally, the Meishan promoter contained a 2-bp deletion within the proximal promoter and a 22-bp deletion within an upstream enhancer region previously identified as important for basal activity of the GnRHR promoter in gonadotropes [30]. Five single bp changes were noted in the comparison of the Index and Control promoters. Jiang and colleagues previously published 1154 bp of porcine GnRHR gene promoter (GenBank AF227685) as part of a QTL study evaluating polymorphisms associated with altered numbers of corpora lutea in European Large White and Meishan breeds [7]; documenting the TG deletion and single bp substitutions at −562 and −845 bp in the Meishan promoter. Further, alignment of the genomic clone Sscrofa 10.2, which is from a mixed breed sample, to the three breeds in this study indicated that the mixed breed sequence was more similar to the Meishan than either the Index or Control. Nine of the 13 observed Meishan polymorphisms aligned with the Sscrofa 10.2 sequence.

**Table 3:** Base changes between lines within the −1915 bp promoters^a^

Line	Change^a^	Location^a^
Meishan	TG deletion^b^	−233/−232
Meishan	C to T^b,c^	−562
Index	A to G	−605
Index	T to C	−651
Meishan	G to T^b^	−845
Index	T to C	−1027
Meishan	T to G	−1094
Index	C to T	−1110
Meishan	A to G^c^	−1211
Meishan	C to G^c^	−1235
Meishan	A to C^c^	−1450
Meishan	C to A^c^	−1615
Meishan	A to G^c^	−1639
Meishan	T to C^c^	−1690
Meishan	22 bp deletion^c^	−1688/−1667
Index	A to T	−1727
Meishan	C deletion^c^	−1738
Meishan	A to G	−1742

^a^Relative to the Control line sequence

^b^These base pair substitutions were also noted in the GenBank sequence AF227685

^c^The Sscrofa 10.2 genomic clone NC_010450.3 had the Meishan base pair substitutions at these locations. At −1219 the genomic clone has a G to A substitution compared to the Control line sequence

### Differential GnRHR promoter activity among genetic lines is not attributable to a SF1 binding site, an essential element to basal activity of the promoter in αT3-1 cells

**Fig. 2:**
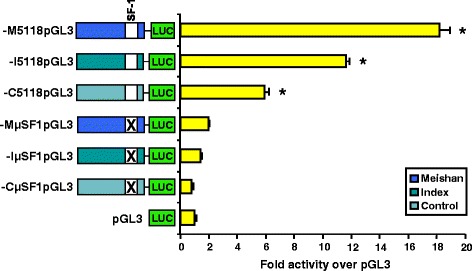
A SF1 binding site is crucial for transcriptional activity of the GnRHR gene in all three genetic lines of swine. Luciferase (LUC) reporter vectors containing block replacement mutation of the SF1 binding site in the context of the full-length promoter were constructed for all three lines of swine (Meishan, −MμSF1pGL3; Index, −IμSF1pGL3; Control, −CμSF1pGL3). These vectors, along with vectors containing full-length native GnRHR promoter for Meishan (−M5118pGL3), Index (−I5118pGL3) and Control (−C5118pGL3) lines of swine were transiently transfected into αT3-1 cells. An asterisk indicates means that are greater than promoterless control (pGL3; *P* < 0.05)

Previously, our laboratory identified a SF1 binding site located −179/−171 upstream of the translational start site as a necessary member of the gonadotrope-specific promoter for the porcine GnRHR gene [30]. Luciferase reporter constructs harboring a block replacement mutation of this gonadotrope specific element (GSE) within the context of the −5118 bp promoter completely blocked luciferase activity compared to vectors containing the −5118 bp promoter alone. Such findings led us to investigate the role of this SF1 binding site with regard to line divergence for GnRHR gene promoter activity. Transient transfection of αT3-1 cells with reporter constructs containing the mutated SF1 binding site within the context of the full-length promoter completely ablated (*P* < 0.05) GnRHR promoter activity for all three lines of pigs (Fig. 2). Therefore, this SF1 binding site is critical for transcriptional activity of the porcine GnRHR promoter in all three genetic lines. However, these data also indicate that this site is not involved in line-dependent activity of the GnRHR gene promoter.

### The region between −1915/−1431 of the porcine GnRHR gene promoter is not alone responsible for line divergent promoter activity

**Fig. 3:**
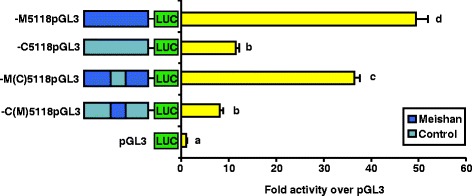
Divergence of GnRHR promoter activity between lines is not alone conferred by the −1915/−1431 bp region. Transient transfection of αT3-1 cells with GnRHR gene promoter “swap” luciferase (LUC) reporter constructs for Meishan and Control lines of swine was performed. The −M(C)5118pGL3 vector is the full-length Meishan reporter construct with the 484 bp region from −1915 to −1431 bp replaced with the Control promoter sequence from that region, whereas the −C(M)5118pGL3 vector is the reverse. Unique letters indicate means different from one another (*P* < 0.05)

Upon sequence comparison of the −1915 promoters for the three lines, we identified a 22 bp deletion (−1682/−1661) accompanied by a single bp substitution (−1684) in the Meishan promoter compared to either the Index or Control promoters. Based on the sequence divergence between the Meishan and Index/Control promoters within the −1915/−1431 region of proximal GnRHR promoter, we performed a promoter “swap” experiment, in which the 484 bp region between −1915/−1431 of the Control and Meishan promoters were swapped within the context of the native full-length promoter. Although luciferase activity of the Meishan promoter decreased (*P* < 0.05) when manipulated to contain 484 bp of the Control promoter, activity of the Meishan GnRHR gene promoter maintained much higher levels than the full-length Control promoter (Fig. 3). Alternatively, activity of the Control promoter containing 484 bp of Meishan promoter sequence remained at significantly lower levels than the full-length Meishan promoter (Fig. 3). Therefore, the −1915/−1431 region does not appear to contain the element(s) responsible for divergence in GnRHR gene promoter activity between Meishan and Index/Control lines of swine.

### Enhanced activity of the Meishan GnRHR gene promoter is diminished upon reduction of the 5’ flanking region to −1004 bp

**Fig. 4:**
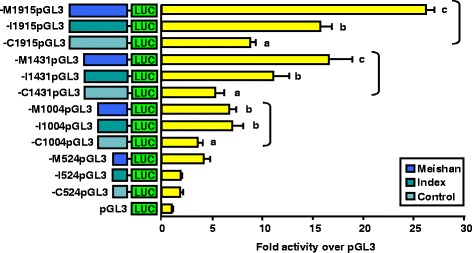
Reduction of the Meishan GnRHR promoter to −1004 bp eliminates its enhanced activity. Luciferase (LUC) reporter vectors containing either 1915, 1431, 1004 or 524 bp of proximal promoter for the GnRHR gene from Meishan, Index and Control lines of swine or promoterless control (pGL3) were transiently transfected into αT3-1 cells. Unique letters within vectors containing the same portion of 5’ flanking sequence (−1915, −1431, −1004 or −524) for each swine line (brackets) indicate values that are significantly different from one another (*P* < 0.05)

We performed transient transfections in αT3-1 cells with plasmids containing sequential 5’ deletions of the −1915 promoter (−1915pGL3, −1431pGL3, −1004pGL3 and −524pGL3) for all three pig lines (Meishan, Index and Control). Removal of approximately 500 bp from the −1915 promoter for Meishan, Index and Control swine maintained genetic line divergence for luciferase activity (Fig. 4). However, reduction of 5’ flanking sequence from −1431 to −1004 eliminated the enhanced promoter activity for the Meishan line (*P* < 0.05) compared to the Index promoter, although luciferase activity for −M1431pGL3 and −I1431pGL3 were still greater (*P* < 0.05) than the corresponding Control promoter (Fig. 4). This suggests the presence of an element(s) partially responsible for line-specific promoter activity within the −1431/−1004 region of the porcine GnRHR gene promoter. We also noted that further reduction of the GnRHR promoters for each line to −524 bp eliminated differences in luciferase activity among the lines (*P* < 0.05), inferring that line-specific elements could reside between −1004/−524.

### A T → C bp substitution at −1690 confers divergent binding between the Meishan and Control/Index GnRHR gene promoters

**Fig. 5:**
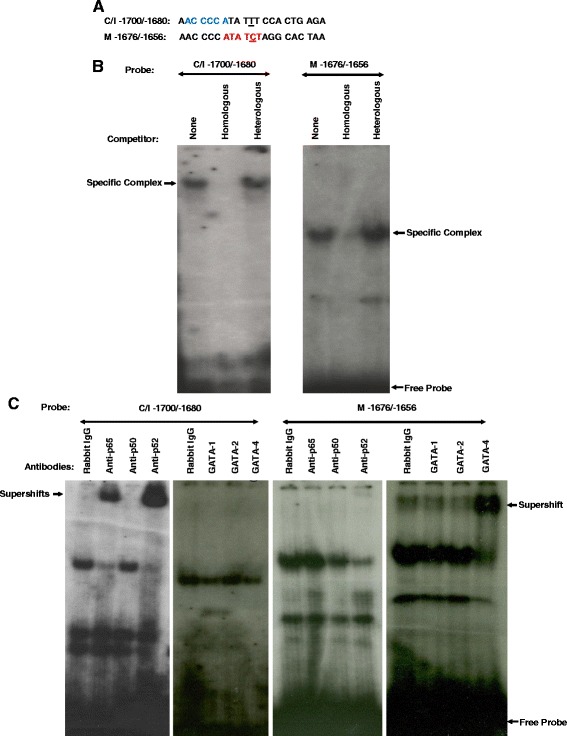
The −1690 bp substitution in the Meishan promoter binds GATA-4, whereas the Control/Index promoters bind NF-κB. **a** Oligonucleotide probes (5’ to 3’) were synthesized containing sequence flanking a naturally occurring point mutation at −1690 of the Control/Index (C/I −1700/−1680) or −1666 of the Meishan (M −1676/−1656) promoter. Underlines within each DNA probe represent the nucleotide substitution between lines and putative NF-κB (ACCCCA; Blue) and GATA-4 (ATATCT; Red) elements identified by sequence analysis are highlighted. **b** EMSAs were performed with αT3-1 nuclear extracts and radiolabeled oligonucleotides spanning the −1690 bp substitution between the Control/Index and Meishan (−1666 bp) swine lines. To determine the specificity of the DNA:protein complex, 50-fold molar excess of either unlabeled homologous or heterologous DNA were added (specific complexes indicated by arrows). **c** To assess which specific proteins comprised the DNA:protein complex binding to the oligonucleotide spanning the substitution at −1690 bp of the Control promoter (−1666 bp of the Meishan promoter), antibodies directed against the p65, p50, and p52 subunits of NF-κB, GATA-1, -2 and -4, or an equal mass of rabbit IgG were added (supershifts indicated by arrows)

Sequence analysis identified a single bp alteration located within the upstream enhancing region at −1690 bp of proximal promoter between the Meishan and homologous Control/Index promoters (Fig. 5a). Electrophoretic mobility shift assays utilizing αT3-1 nuclear extracts and radiolabeled oligonucleotides spanning the bp substitution (−1690) between the Meishan and Control/Index promoters revealed a specific binding complex for both swine lines (Fig. 5b). Sequence analysis of this region identified potential NF-κB and GATA elements in the Meishan and Control/Index promoters. Addition of antibodies directed against the p52 and p65 subunits of NF-κB resulted in a supershift of the DNA:protein complex for the Control/Index oligonucleotide (Fig. 5c). However, the specific binding complex associated with the Meishan oligonucleotide was supershifted by an antibody specific for GATA-4 (Fig. 5c). Therefore, the single bp alteration in the Meishan promoter forms a GATA element that allows GATA-4 binding instead of the p52 and p65 subunits of NF-κB which bind to the Control/Index promoters.

### Block replacement mutations of both the GATA-4 and NF-κB binding sites in the Meishan and Control/Index promoters, respectively, attenuated luciferase activity

**Fig. 6:**
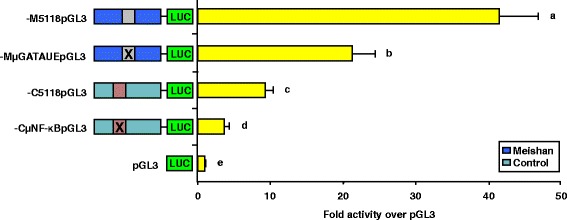
Mutation of GATA-4 and NF-κB elements indicate those sites are functionally relevant to promoter activity. To confirm the importance of the GATA-4 binding site within the Meishan promoter and the NF-κB element within the Control/Index promoters, block replacement mutations of each binding site were constructed within the context of their respective full length promoter. Transient transfection of αT3-1 cells with luciferase (LUC) reporter vectors containing both full-length promoters (−M5118pGL3 and −C5118pGL3), block replacement mutations of the GATA-4 (−MμGATAUEpGL3) and NF-κB (−CμNF-κBpGL3) elements or promoterless control (pGL3) were performed. Differences between vectors are indicated by bars with unique letters (*P* < 0.05)

Transient transfections of αT3-1 cells with luciferase reporter vectors containing either the native, full-length Meishan (−M5118pGL3) and Control (−C5118pGL3) promoters, block replacement mutations of the GATA-4 (−MμGATAUE-4pGL3) and NF-κB (−CμNF-κBpGL3) binding sites within the context of their respective full-length promoter or promoterless control (pGL3) were performed. Reporter vectors containing the block replacement mutation of the GATA-4 binding site (−MμGATAUEpGL3) resulted in approximately a 50 % reduction in luciferase activity (*P* < 0.05) compared to the native Meishan promoter (Fig. 6). Further, αT3-1 cells transfected with the reporter vectors containing the block replacement mutation of the NF-κB binding site (−CμNF-κBpGL3) reduced luciferase activity (*P* < 0.05) by approximately 60 % compared to cells containing the native Control promoter (Fig. 6). Thus, this single bp substitution contributes to the line-specific activity of the porcine GnRHR gene promoter.

### A C → G bp substitution at −1235 promotes binding of transcription factors unique to the Meishan GnRHR promoter

**Fig. 7:**
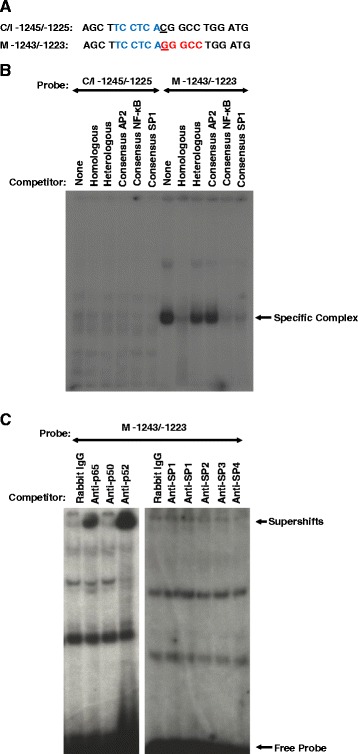
A bp substitution at −1235 allows for NF-κB and SP1-like factor binding to the Meishan promoter. **a** Oligonucleotide probes (5’ to 3’) were synthesized containing sequence flanking a naturally occurring point mutation at −1235 of the Control/Index (C/I −1245/−1225) or −1232 of the Meishan (M −1243/−1223) promoter. Underlines within each DNA probe represent the nucleotide substitution between lines and putative NF-κB (TCCTCA; Blue) and SP1 (GGCGG; Red) elements identified by sequence analysis are highlighted. **b** EMSAs were performed by incubating radiolabeled oligonucleotides with nuclear extracts (5 μg) from αT3-1 cells and specificity of DNA-protein interactions was assessed by competition with 50-fold molar excess of homologous or heterologous unlabeled DNA (specific complexes indicated by arrows). Additionally, the DNA-protein complex was challenged by competition with 50-fold molar excess of unlabeled oligonucleotides containing consensus binding sequences for AP2, NF-κB and SP1. Electrophoresis of the gel was performed for an extended time, thus, free probe was run off the gel. **c** To determine the specific factors comprising the Meishan-specific complex, nuclear extracts were also incubated with antibodies directed against the p50, p52, and p65 subunits of NF-κB, SP1 (Upstate), SP1 (Santa Cruz Biotechnology), SP2, SP3, SP4 or an equal mass of rabbit IgG (supershift indicated by arrows)

**Fig. 8:**
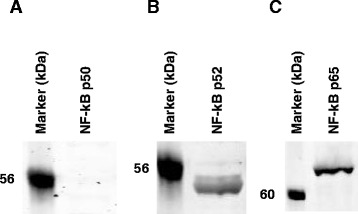
The NF-κB subunits p52 and p65, but not p50 are present in αT3-1 nuclear extracts. Western blot analysis was performed with αT3-1 nuclear extracts (40 μg) and antibodies specific for the **a** p50 **b** p52 and **c** p65 subunits of NF-κB as described in Materials and Methods

Sequence analysis of the −1431/−1004 region of proximal promoter revealed five single-bp substitutions among the swine lines. EMSAs using radiolabeled oligonucleotides spanning each of these substitutions (data not shown) revealed that the C → G alteration at −1235, relative to the translational start site (Fig. 7a), allowed binding of nuclear proteins from αT3-1 cells to the oligonucleotide from the Meishan promoter, whereas the oligonucleotide representing the Control/Index promoter failed to bind protein (Fig. 7b). Competition with oligonucleotides composed of the consensus binding sequences for AP2, NF-κB, and SP1 resulted in ablation of binding by the NF-κB and SP1 consensus oligonucleotides (Fig. 7b). Inclusion of antibodies directed against the p52 and p65 subunits of NF-κB in EMSAs with the oligonucleotide containing the bp substitution at −1235 bp of the Meishan GnRHR gene promoter revealed a supershift of the DNA-protein complex, whereas the addition of an antibody specific for the p50 subunit of NF-κB did not affect complex migration (Fig. 7c). Further, addition of antibodies directed against SP1 (from two separate commercial vendors), SP2, SP3 and SP4 did not result in a supershift (Fig. 7c). Western blot analysis of αT3-1 nuclear extracts confirmed that the p52 and p65 subunits of NF-κB are present, whereas no protein was detected for the p50 subunit (Fig. 8). Thus, the p52 and p65 subunits of NF-κB are members of the protein complex binding to the −1240/−1230 region of the Meishan promoter. In addition, a factor capable of binding to the consensus SP1 oligonucleotide is likely another member comprising the Meishan-specific binding complex within this promoter region.

### Block replacement mutations of the NF-κB and SP1 binding sites located between −1240/−1230 of 5’ flanking region reduce activity of the Meishan GnRHR promoter

**Fig. 9:**
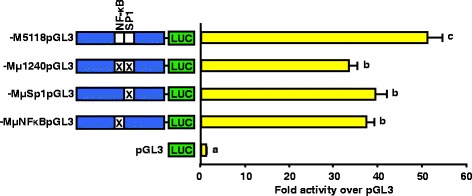
Mutations of the NF-κB, SP1 or both elements reduce activity of Meishan reporter constructs. Luciferase (LUC) reporter vectors containing either the native, full-length Meishan GnRHR gene promoter (−M5118pGL3), block replacement mutations of the NF-κB (−MμNF-κBpGL3), SP1 (−MμSp1pGL3) or a combination of the two (−Mμ1240pGL3) binding sites within the context of −M5118pGL3, or promoterless control (pGL3) were transiently transfected into αT3-1 cells. Unique letters indicate means that are significantly different from one another (*P* < 0.05)

Luciferase reporter vectors containing block replacement mutations of the binding elements for NF-κB (−MμNF-κBpGL3), SP1 (−MμSP1pGL3), or both (−Mμ1240pGL3) within the context of the full-length promoter from the Meishan line were transiently transfected into αT3-1 cells to determine the importance of these binding elements to overall activity of the Meishan GnRHR promoter. All three vectors containing block replacement mutations reduced luciferase activity (*P* < 0.05) approximately 25 % when compared to the vector containing the native full-length Meishan promoter (Fig. 9). Despite the decreased luciferase activity for the vectors containing block replacement mutations, they appear higher (33- to 39-fold over promoterless control) than the values obtained for the vector containing the full-length Control promoter (−C5118pGL3) in Fig. 1 (19-fold over promoterless control). This suggests that while this region confers increased promoter activity of the Meishan GnRHR promoter, it acts synergistically with other elements(s) located within the Meishan proximal promoter.

### A third bp substitution (G → T) located at −845 bp confers divergent GATA-4 binding to the Meishan GnRHR promoter

**Fig. 10:**
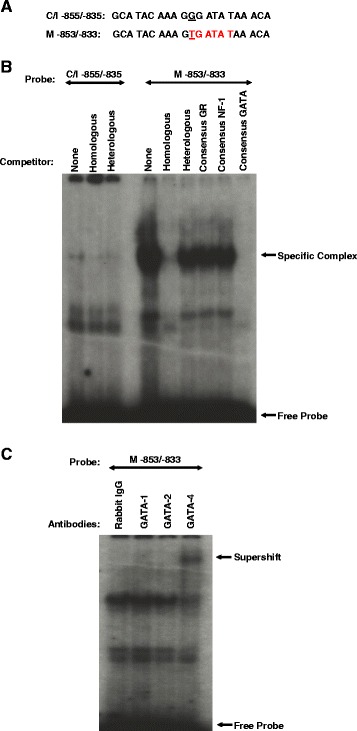
A bp substitution (G → T) located at −845 of the Meishan promoter allows GATA-4 to bind. **a** Oligonucleotide probes (5’ to 3’) were synthesized containing sequence flanking a naturally occurring point mutation at −845 of the Control/Index (C/I −855/−835) and −843 of the Meishan (M −853/−833) promoter. Underlines within each DNA probe represent the nucleotide substitution between lines and the putative GATA element (TGATAT; Red) identified by sequence analysis is highlighted. **b** EMSAs were performed by incubating radiolabeled oligonucleotides with nuclear extracts (5 μg) from αT3-1 cells and specificity of DNA-protein interactions was assessed by competition with 50-fold molar excess of homologous or heterologous unlabeled DNA (specific complexes indicated by arrows). Additionally, the DNA-protein complex was challenged by competition with 50-fold molar excess of unlabeled oligonucleotides containing consensus binding sequences for GR, NF-1 and GATA. **c** To determine the specific factor(s) comprising the Meishan-specific complex, nuclear extracts were also incubated with antibodies directed against GATA-1, -2 and -4 or an equal mass of rabbit IgG (supershift indicated by arrows)

Examination of the sequence between −1004/−524 of proximal promoter indicated four additional sequence differences when comparing the three lines of swine. To determine if these single-bp substitutions resulted in binding differences among lines, EMSAs were performed with radiolabeled oligonucleotides spanning the bp alterations. A bp substitution (G → T) located at −845 (Fig. 10a), relative the translational start site, resulted in binding of nuclear extracts from αT3-1 cells for the Meishan but not the Control/Index oligonucleotides (Fig. 10b). The specific complex was abrogated by addition of unlabeled oligonucleotide containing consensus binding sites for GATA (Fig. 10b). With the addition of antibodies specific for GATA-1, GATA-2, and GATA-4 or an equal mass of rabbit IgG, a supershift of the complex was observed with the GATA-4 antibody (Fig. 10c). Thus, a single-bp substitution (G → T) at −845 bp of the Meishan GnRHR promoter results in formation of a GATA-4 binding site.

### Block replacement mutation of the GATA-4 binding site at −845/−840 of 5’ flanking region reduces activity of the Meishan GnRHR promoter

**Fig. 11:**
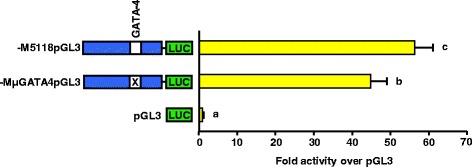
Mutation of the Meishan-specific GATA-4 binding site diminished promoter activity in αT3-1 cells. Cells were transiently transfected with luciferase (LUC) vectors containing either the native, full-length Meishan promoter (−M5118pGL3), a block replacement mutation of the GATA-4 binding site within the context of −M5118pGL3 (−MμGATA4pGL3), or promoterless control (pGL3). Unique letters indicate means that are significantly different from one another (*P* < 0.05)

Luciferase reporter vectors were constructed containing a block replacement mutation of the GATA-4 binding site within the context of the full-length Meishan promoter (−MμGATA4pGL3). Transient transfections were performed in the αT3-1 cell line with reporter vectors containing the full-length Meishan promoter (−M5118pGL3), −MμGATA4pGL3, and promoterless control (pGL3). The GATA-4 block replacement mutation reduced luciferase activity (*P* < 0.05) approximately 20 % compared to the full-length Meishan promoter (Fig. 11). Thus, the enhanced activity of the Meishan GnRHR gene promoter is partially due to this unique GATA-4 binding site. Consistent with our previous results, the values obtained for the −MμGATApGL3 vector (45-fold over promoterless control) were higher than those established for the full-length Control promoter (19-fold over promoterless control; Fig. 1). Therefore, this element, along with the two already described (NF-κB and SP1), do not fully explain the divergent Meishan GnRHR promoter activity.

## Discussion

Here, we have demonstrated a dramatic variance in activity among reporter vectors containing GnRHR promoters from the Control, Index and Meishan lines of pigs using transient transfections in the gonadotrope-derived αT3-1 cell line. Divergent activity of the Control, Index, and Meishan promoters illustrates a potential alteration in the mechanisms underlying transcriptional regulation of the porcine GnRHR gene among genetic strains. Ultimately, differential regulation of GnRHR gene expression may be correlated with divergent ovulation rates as well as other reproductive traits observed among the Control, Index and Meishan pig lines. This characteristic difference in activity among pig strains was lost after promoter constructs were reduced from −1431 to −1004 bp of 5’ flanking region, inferring that the element(s) responsible for the elevated luciferase activity of the Meishan promoter construct is located within the proximal 1431 bp of GnRHR promoter. Within gonadotrope-derived cell lines, the majority of elements responsible for basal and hormonally-induced expression of the GnRHR gene in other species are located within 1000 bp of proximal promoter [10–19, 22–28, 36, 37], although placental-, granulosa/luteal cell- and neuronal-specific promoters have been identified further upstream in the human [38–40]. On the other hand, the spatial arrangement of the porcine GnRHR promoter is somewhat unique because it requires approximately 1800 bp of 5’ flanking sequence for basal activity in αT3-1 cells [30]. Despite the fact that the elements which confer line-specific activity of the porcine GnRHR promoter lie further upstream than 1000 bp, they still remain within the boundaries of the gonadotrope-specific promoter.

Transcriptional regulation of the GnRHR gene in different species is achieved through a variety of mechanisms and a number of different transcription factors [8, 9]. One of the most characterized mechanisms for transcriptional regulation of the GnRHR gene involves the orphan nuclear receptor, SF1, known to be vital for gonadotrope-specific expression of the GnRHR gene in the human [26], mouse [11], rat [24], sheep [29] and pig [30]. SF1 binding is also known to confer expression of the gonadotropin subunit genes within gonadotrope cells [41–43]. Previously, our laboratory demonstrated that three SF1 binding sites are involved in transcriptional regulation of the porcine GnRHR gene promoter in αT3-1 cells. In reporter assays, mutation of the proximal SF1 binding site at −179/−171 bp resulted in complete ablation of promoter activity, indicating that this site is essential for gene expression [30]. In this study, however, we have shown that the critical proximal SF1 binding site is not responsible for line-specific regulation of the GnRHR gene in swine (Fig. 2).

Identification of an NF-κB site involved in line-specific expression of the porcine GnRHR gene in gonadotrope cells is unique. While NF-κB has traditionally been associated with genes involved in immune and inflammatory responses [44], it has been implicated in mediating the apoptotic effects of GnRH in ovarian cancer cells [45]. As reviewed by Hayden and Ghosh [46], NF-κB typically exists as a heterodimer composed of p65 (RelA), p50, p52, RelB and/or c-Rel. Although p52/p65 and p52/RelB heterodimers are prevalent, most commonly the heterodimer is comprised of the p65 and p50 subunits. Previously we reported that p52/p65 subunits are involved in the transcriptional regulation of the porcine GnRHR2 gene in the testis [47]. Regarding gonadotrope cells of the anterior pituitary gland, however, NF-κB remains noticeably absent from the cast of transcription factors known to regulate the GnRHR or gonadotropin subunit genes. In this study, we detected p52 and p65 proteins within αT3-1 nuclear extracts and established the role of p52/p65 heterodimer binding in transcriptional regulation of the GnRHR gene. Consistent with this, NF-κB is an important regulator of Cox-2 promoter activity in the gonadotrope-derived cell line, LβT2, and treatment with GnRH stimulated phosphorylation of the p65 subunit by 22-fold [48]. In addition, NF-κB binding sites have also been associated with regulation of genes expressed in other pituitary cell types including somatotropes [49] and corticotropes [50].

The −1240/−1230 region of the Meishan GnRHR promoter also appears to bind another transcription factor capable of interacting with the SP1 consensus binding site. Although the use of an SP1 consensus oligonucleotide revealed competition for DNA-protein binding (Fig. 4), the inability of SP1-specific antibodies acquired from two separate commercial vendors to bind the DNA-protein complex (Fig. 4) suggests that an alternative transcription factor recognizes the SP1 consensus binding sequence. To further verify the integrity of the antibodies directed against SP1, we also performed EMSAs with αT3-1 nuclear extracts and radiolabeled oligonucleotide containing consensus binding sites for SP1. In this instance, a specific complex was formed and the addition of SP1-specific antibodies resulted in supershifted DNA-protein complexes (data not shown), confirming the presence of SP1 in nuclei of αT3-1 cells and effectiveness of the SP1 antibodies. Another concern was whether the SP1 consensus oligonucleotides actually contained one or more NF-κB elements and therefore, was merely mimicking the NF-κB consensus oligonucleotide. However, sequence analysis of the oligonucleotide containing consensus SP1 binding sites did not reveal any NF-κB elements. Next, we examined other members of the SP1 family of transcription factors. Of primary interest, SP3 and SP4 recognize the same binding sequence with similar affinities [51]. However, the addition of antibodies directed against SP2, SP3 and SP4 were unable to bind to the specific complex. Thus, we were able to eliminate SP1-4 as potential transcription factors binding to the SP1 element located within −1240/−1230 of the Meishan GnRHR promoter. Currently, nine members of the SP1 family (SP1-9) have been identified [52, 53], and represent a subgroup of a larger class of transcription factors, the SP1-like/Krüppel-like factor (KLF) family [54]. These factors share a highly conserved DNA-binding domain containing three Cys^2^/His^2^ zinc fingers [51], which is the most abundant transcription factor motif in the human genome [55]. In fact, over 25 SP1-like/KLF genes have been reported in mammals [56]. The nine SP transcription factors can further be divided into the SP1-like family (SP1-4) and SP8-like family (SP5-9) [57]. While SP1 and SP3 are ubiquitously expressed, SP5-9 expression patterns are more specific and temporal. SP5 and SP8 expression has been linked with Wnt activity and are important for stem cell differentiation and early embryonic development [58, 59]. SP6, also known as epiprofin, is involved in epidermal differentiation [60] whereas SP7, known as osterix, is expressed in osteoblasts [61]. Additionally, SP9 is also expressed embryonically and affects limb outgrowth [62]. Given the developmental roles that SP5-9 frequently play, it is unlikely that any of these factors are binding the GnRHR gene promoter. Thus, more studies are required to determine the identity of the factor(s) binding to the SP1 site within the −1240/−1230 promoter region of the Meishan GnRHR gene.

Mutation of the individual binding sites for NF-κB and the SP1-like factor within the Meishan GnRHR promoter demonstrated a significant loss of promoter activity. In addition, mutation of both the NF-κB and the SP1 recognition sequences (−Mμ1240pGL3) diminished luciferase activity to approximately the same level as either single block mutation. Due to the lack of further reduction of luciferase activity by the double block replacement mutation, it would appear that the transcription factors binding to the two elements work synergistically, but not additively, to stimulate increased GnRHR promoter activity in the Meishan line of swine. Indeed, NF-κB interacts with a variety of other transcription factors including AP1, estrogen receptor α, C/EBP, SF1 and SP1 [63–66]. The Nabel laboratory determined that SP1 interacts with p65 via the DNA binding regions of each factor and that this interaction is necessary for activation of the HIV-1 gene [67]. Despite the importance of the complex of transcription factors binding at −1240/−1230, none of the mutations lowered Meishan GnRHR promoter activity to that of the native full-length Control promoter suggesting another element(s) within the Meishan GnRHR promoter also confers line-specific expression.

We also identified 2 additional single-bp alterations located at −845 (G → T) and at −1690 (T → C) within the Meishan GnRHR promoter region that allowed binding of GATA-4 to the recognition sites. While GATA-4 has not previously been implicated in transcriptional regulation of the GnRHR gene, it is involved in the expression of other gonadotropic genes. A GATA motif detected within the human α-subunit gene promoter binds GATA-2 and a GATA-4-related protein in αT3-1 cells [68]. These investigators were unable to confirm GATA-4 binding because specific antibodies directed against GATA-4 were not commercially available at the time. Consistent with these results, our study confirmed the presence of GATA-4 in gonadotrope-derived αT3-1 cells and implicated its importance in regulation of gonadotropic gene expression. Steger and coworkers [68] also reported that the same GATA element binds GATA-2 and -3, but not GATA-4-related protein, in placental-derived cell lines. Binding of GATA-4 also regulates other genes essential to reproduction [69]. In the neuronal GT1-7 cell line, GATA-4 binds recognition sites within the promoter for the GnRH gene [70]. Transcription of the Müllerian inhibiting substance (MIS) gene in Sertoli cells is enhanced by the direct interaction of GATA-4 and SF1, although GATA-4 binding to the DNA is not required for this synergistic effect [71, 72]. Additionally, adrenal-specific transcription of the human P450c17 gene is regulated by the interaction of GATA-4 or GATA-6 with SP1 [73]. However, despite its contribution to enhanced promoter activity of the Meishan GnRHR gene, the GATA-4 binding sites, like the NF-κB and SP1 elements, do not fully explain the increased activity of reporter constructs containing the Meishan compared to Control/Index promoters. Therefore, future studies in our laboratory will focus on identifying the remaining elements and corresponding binding factors that contribute to the enhanced activity of the Meishan GnRHR promoter in αT3-1 cells.

## Conclusions

**Fig. 12:**
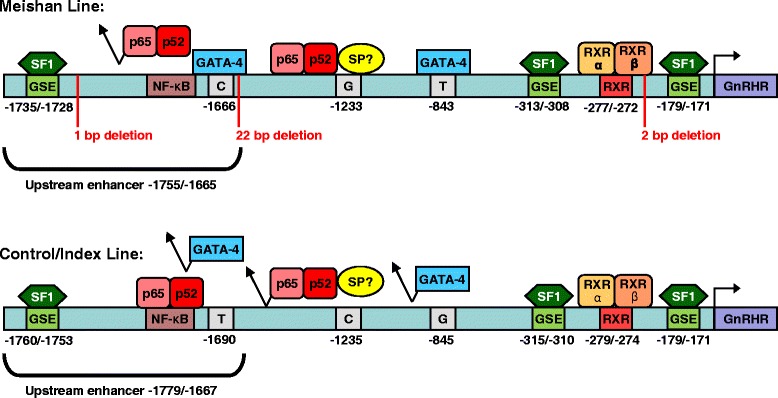
Diagrammatic model of the GnRHR gene promoters from the Meishan and Control/Index lines of swine. Within the upstream enhancer region, a bp substitution (T → C) next to the 22-bp deletion results in a functional GATA-4 site in the Meishan promoter, however, a neighboring NF-κB site in the control promoter binds the p52 and p65 subunits of NF-κB in the absence of GATA-4 binding. A single-bp substitution (C → G) at −1232 in the Meishan compared to Control/Index (−1235) GnRHR gene promoters allows for binding of a complex of transcription factors including the p52 and p65 subunits of NF-κB and a SP1-like protein capable of binding to a SP1 binding site. Interestingly, the NF-κB and SP1 elements within the Control/Index promoter overlap by a single bp, preventing binding of the protein complex, whereas the polymorphism (C → G) specific to the Meishan promoter shifts the SP1 site 3’ by 1 bp allowing it to abut the NF-κB element. An additional G → T bp alteration at −845 of the porcine GnRHR gene allows for Meishan-specific binding of GATA-4. An RXR binding site at −279/−274 and three SF1 binding sites (GSE) at −179/−171, −315/−310 and −1760/−1753 are members of the gonadotrope-specific promoter for the porcine GnRHR gene [30] and therefore, common to both lines

In conclusion, the large variance in GnRHR promoter activity among the Control, Index, and Meishan lines of swine appears to be due, in part, to binding of the p52 and p65 subunits of NF-κB and a SP1-like transcription factor that recognizes an SP1 binding sequence within the Meishan versus Control and Index promoters (Fig. 12). The NF-κB and SP1 elements within the Control/Index promoter overlap by a single bp (−1235), preventing binding of the protein complex. However, the polymorphism (C → G) specific to the Meishan promoter shifts the SP1 site 3’ by 1 bp, allowing it to abut with the NF-κB element, thereby conferring binding of the protein complex to the recognition sequences. Another polymorphism (G → T) located at −845 of the Meishan GnRHR promoter binds GATA-4, enhancing its activity in αT3-1 cells compared to the Control or Index promoters (Fig. 12). An additional distal polymorphism (−1690) within the upstream enhancer allows GATA-4 to bind to the Meishan promoter, whereas the p52 and p65 subunits of NF-κB bind to the homologous Control/Index promoters (Fig. 12). Therefore, the 3 identified elements unique to the Meishan promoter contribute to its enhanced activity over the Control/Index promoters. These data illustrate the alternative mechanisms employed by the Meishan line of swine to regulate GnRHR gene expression in gonadotropes. To our knowledge, this represents the first report of genetic polymorphisms within the 5’ flanking sequence of the GnRHR gene that directly result in divergent promoter activity. Furthermore, we demonstrated a unique role for the NF-κB family of transcription factors in transcriptional regulation of the GnRHR gene within gonadotropes of the anterior pituitary gland.

## Abbreviations

AP1, activator protein 1; CRE, cAMP responsive element; EMSA, electrophoretic mobility shift assay; FOXL2, forkhead box L2; FSH, follicle stimulating hormone; GnRH, gonadotropin-releasing hormone; GnRHR, gonadotropin-releasing hormone receptor; GnSE, GnRHR specific enhancer; GRAS, GnRH receptor activating sequence; GSE, gonadotrope specific element; JNK, c-Jun N-terminal kinase; LH, luteinizing hormone; LHX3, LIM homeobox 3; NF-Y, nuclear factor Y; NF-κB, nuclear factor-kB; OCT1, octamer transcription factor 1; PCR, polymerase chain reaction; Pitx-1, pituitary homeobox 1; PKA, protein kinase A; PKC, protein kinase C; RSV, Rous sarcoma virus; RXR, retinoid X receptor; SAP, SF1 adjacent protein; SF1, steroidogenic factor 1; SP, specificity protein; SURG-1, sequence underlying responsiveness to GnRH

## Additional file

Additional file 1: Raw transfection data. (XLS 90 kb)
