# Erratum: Divergent activity of the gonadotropin-releasing hormone receptor gene promoter among genetic lines of pigs is partially conferred by nuclear factor (NF)-kB, specificity protein (SP)1-like and GATA-4 binding sites

**DOI:** 10.1186/s12958-016-0172-y

**Published:** 2016-07-25

**Authors:** Emily A. McDonald, Jacqueline E. Smith, Rebecca A. Cederberg, Brett R. White

**Affiliations:** Laboratory of Reproductive Biology, Department of Animal Science, Institute of Agriculture and Natural Resources, University of Nebraska-Lincoln, Lincoln, NE USA; Present address: Center for International Health Research, Rhode Island Hospital, Providence, RI USA; Present address: Stowers Institute for Medical Research, Kansas City, MO USA

## Update and Erratum

The title for the original version of this article [1] unfortunately contained an error. ‘(NF)-B’ has been corrected to ‘(NF)-kB’.
